# Robot-Assisted Rehabilitation of Ankle Plantar Flexors Spasticity: A 3-Month Study with Proprioceptive Neuromuscular Facilitation

**DOI:** 10.3389/fnbot.2016.00016

**Published:** 2016-11-14

**Authors:** Zhihao Zhou, Yao Sun, Ninghua Wang, Fan Gao, Kunlin Wei, Qining Wang

**Affiliations:** ^1^The Robotics Research Group, College of Engineering, Peking University, Beijing, China; ^2^Beijing Innovation Center for Engineering Science and Advanced Technology (BIC-ESAT), Peking University, Beijing, China; ^3^Rehabilitation Neuroscience Laboratory, University of Victoria, Victoria, BC, Canada; ^4^Department of Rehabilitation Medicine, First Hospital, Peking University, Beijing, China; ^5^Department of Health Care Sciences, University of Texas Southwestern Medical Center, Dallas, TX, USA; ^6^Motor Control Laboratory, Department of Psychology, Peking University, Beijing, China

**Keywords:** ankle spasticity, plantar flexors, proprioceptive neuromuscular facilitation, robot-assisted rehabilitation, stroke

## Abstract

In this paper, we aim to investigate the effect of proprioceptive neuromuscular facilitation (PNF)-based rehabilitation for ankle plantar flexors spasticity by using a Robotic Ankle–foot Rehabilitation System (RARS). A modified robot-assisted system was proposed, and seven poststroke patients with hemiplegic spastic ankles participated in a 3-month robotic PNF training. Their impaired sides were used as the experimental group, while their unimpaired sides as the control group. A robotic intervention for the experimental group started from a 2-min passive stretching to warming-up or relaxing the soleus and gastrocnemius muscles and also ended with the same one. Then a PNF training session including 30 trials was activated between them. The rehabilitation trainings were carried out three times a week as an addition to their regular rehabilitation exercise. Passive range of motion, resistance torque, and stiffness were measured in both ankles before and after the interventions. The changes in Achilles tendon length, walking speed, and lower limb function were also evaluated by the same physician or physiotherapist for each participant. Biomechanical measurements before interventions showed significant difference between the experimental group and the control group due to ankle spasticity. For the control group, there was no significant difference in the 3 months with no robotic intervention. But for the experimental group, passive dorsiflexion range of motion increased (*p* < 0.01), resistance torque under different dorsiflexion angle levels (0°, 10°, and 20°) decreased (*p* < 0.05, *p* < 0.001, and *p* < 0.001, respectively), and quasi-static stiffness under different dorsiflexion angle levels (0°, 10°, and 20°) also decreased (*p* < 0.01, *p* < 0.001, and *p* < 0.001, respectively). Achilles’s tendon length shortened (*p* < 0.01), while its thickness showed no significant change (*p* > 0.05). The robotic rehabilitation also improved the muscle strength (*p* < 0.01) and muscle control performance (*p* < 0.001). In addition, improvements were observed in clinical and functional measurements, such as Timed Up-and-Go (*p* < 0.05), normal walking speed (*p* > 0.05), and fast walking speed (*p* < 0.05). These results indicated that the PNF-based robotic intervention could significantly alleviate lower limb spasticity and improve the motor function in chronic stroke participant. The robotic system could potentially be used as an effective tool in poststroke rehabilitation training.

## 1 Introduction

In China, Cerebrovascular Accident (CVA) has exceeded heart disease to become the leading cause of death and adult disability (Chen, 2008). There are more than 2.5 million new stroke cases each year in China alone, which is equivalent to approximately one stroke every 13 s (Liu et al., 2011). Ankle spasticity is one of the most common movement disorders after CVA (O’dwyer et al., 1996). Spastic hypertonic ankles can severely weaken the mobility and the independence of stroke survivors (Winters et al., 1987; Harlaar et al., 2000). Several studies show that limb spasticity is mostly caused by hyperactive reflexes (Rack et al., 1984; Thilmann et al., 1991; Meinders et al., 1996), while others believe that spastic hypertonia is dependent of non-reflex biomechanical change of muscle and connective tissues (Lee et al., 1987; Dietz et al., 1991; Sinkjaer et al., 1996). Although the underlying mechanism of the spasticity is not very clear (Krebs et al., 2003), increased resistance in spastic limb movements can often be observed.

Robotic rehabilitation has been proposed to support physicians so that it can transform the rehabilitation process from labor-intensive tasks to robot-assisted tasks. It can also facilitate quantitative diagnosis, provide customized therapies, and maintain medical records (Krebs et al., 2003; Stegall et al., 2013; Zhang et al., 2013). Currently, several robotic ankle rehabilitation systems have been developed by different research groups, which can be roughly divided into two categories: powered mobile systems and platform-based systems (Saglia et al., 2013; Zhang et al., 2013). Powered mobile ankle systems generally refer to active ankle–foot orthoses (AFOs), mainly focusing on improving walking gaits, e.g., Blaya and Herr (2004); Bharadwaj et al. (2005); Ferris et al. (2006); Roy et al. (2007); Noël et al. (2008). They are usually worn on the foot and provide direct assistance to ameliorate abnormal gait patterns (Shorter et al., 2013). Different from that, a platform-based system is attached to the user’s foot and to a stationary reference like the floor, which mainly aims to improve ankle joint performance (Zhang et al., 2002; Deutsch et al., 2007; Homma and Usuba, 2007; Cordo et al., 2008; Burdea et al., 2013).

Most of the studies mentioned above indicate that the primary work is to alleviate the spasticity of crus muscle for the spastic ankle joint.

For people who have moderate or severe ankle spasticity, a platform-based system is usually applied before powered mobile system to ensure that the gait training is safe and effective. In clinic, continuous passive motion (CPM) is mostly applied in platform-based devices (Singer et al., 2002; Zhang et al., 2002; Selles et al., 2005; Homma and Usuba, 2007; Gao et al., 2011). Singer et al. (2002) examined changes in muscle length and resistance to passive lengthening in the triceps surae muscles in patients with recent brain injury. An intelligent CPM stretching device was developed in Zhang et al. (2002) to treat ankles of neurologically impaired patients. In Selles et al. (2005), stretching of the plantar- and dorsiflexors of the ankle is three times a week for 45 min during a 4-week period. Significant improvements were found in the passive range of motion (ROM), maximum voluntary contraction, ankle stiffness, and comfortable walking speed. In Gao et al. (2011), ten stroke survivors with ankle spasticity/contracture received intervention of 60-min ankle stretching. Stretching significantly improved the force output of the impaired calf muscles in stroke survivors under matched stimulations, and ankle ROM was also increased by stretching. Homma and Usuba (2007) also developed a CPM-based device but did not present the clinical experiments. However, improvements of muscle strength and coordination are limited due to the lack of voluntary movement (Maynard et al., 2005; Zhou et al., 2015). Moreover, there are few studies which focus on long-term robotic treatment (always less than 6-week) for ankle joint with plantar flexor spasticity.

To address these problems, we have integrated a neurophysiological method: proprioceptive neuromuscular facilitation (PNF) technique, into a robotic ankle–foot rehabilitation system (PKU-RARS, short for Robotic Ankle–foot Rehabilitation System, Peking University) and verified the robotic system and treatment method via some preliminary experiments (Zhou et al., 2014, 2015). In this study, we develop a modified version of the device to solve some drawbacks of the previous version according to our existing clinical trials and feedback from patients and therapists. On this modified platform, we try to further investigate effects of long-term robotic PNF rehabilitation on ankle plantar flexors spasticity via more comprehensive outcome evaluation. Seven poststroke patients were recruited and participated in 3-month interventions. Biomechanical, clinical, and functional evaluations were performed before and after the training period, including passive and active biomechanical properties of ankle joint, Achilles’s tendon (AT) properties, and 10-m normal and fast walking.

We introduce the hardware of the proposed RARS and its modifications in Section 2. PNF-based rehabilitation therapy, participants, experiment protocol, and outcome analysis are reported in Section 3. Results are presented and discussed in Sections 4 and 5, respectively, and we conclude in Section 6.

## 2 Robotic Ankle–Foot Rehabilitation System

**Figure 1:**
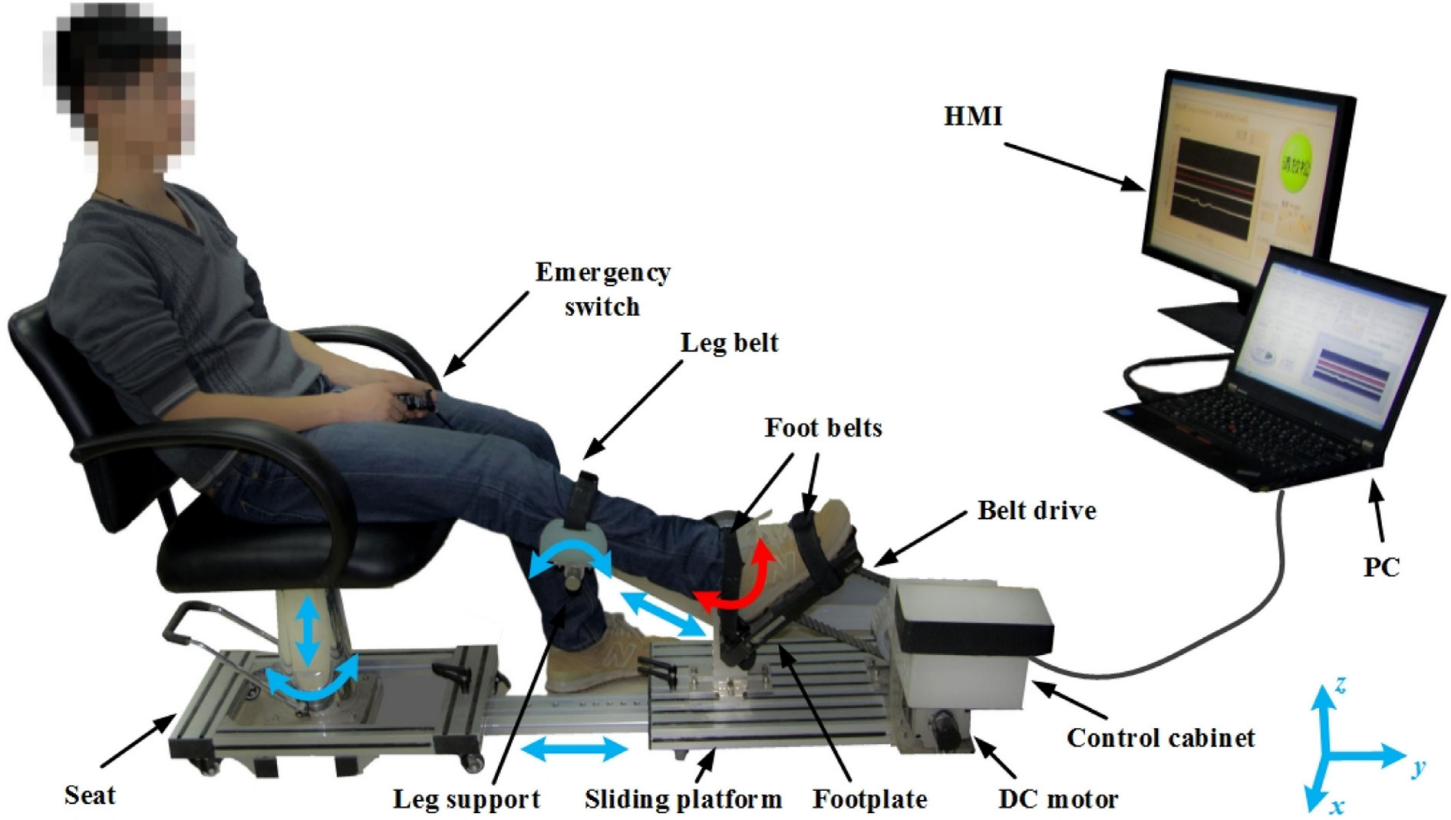
**Overview of the proposed PKU-RARS**. It consists of an adjustable seat, a sliding platform, a leg support, a robotic footplate, an actuator, and a control cabinet, two handheld emergency switches, and a human–machine interface (HMI). The blue arrows represent the passive adjustable degrees of freedom, while the red arrow represents the active movement of the ankle joint.

**Figure 2:**
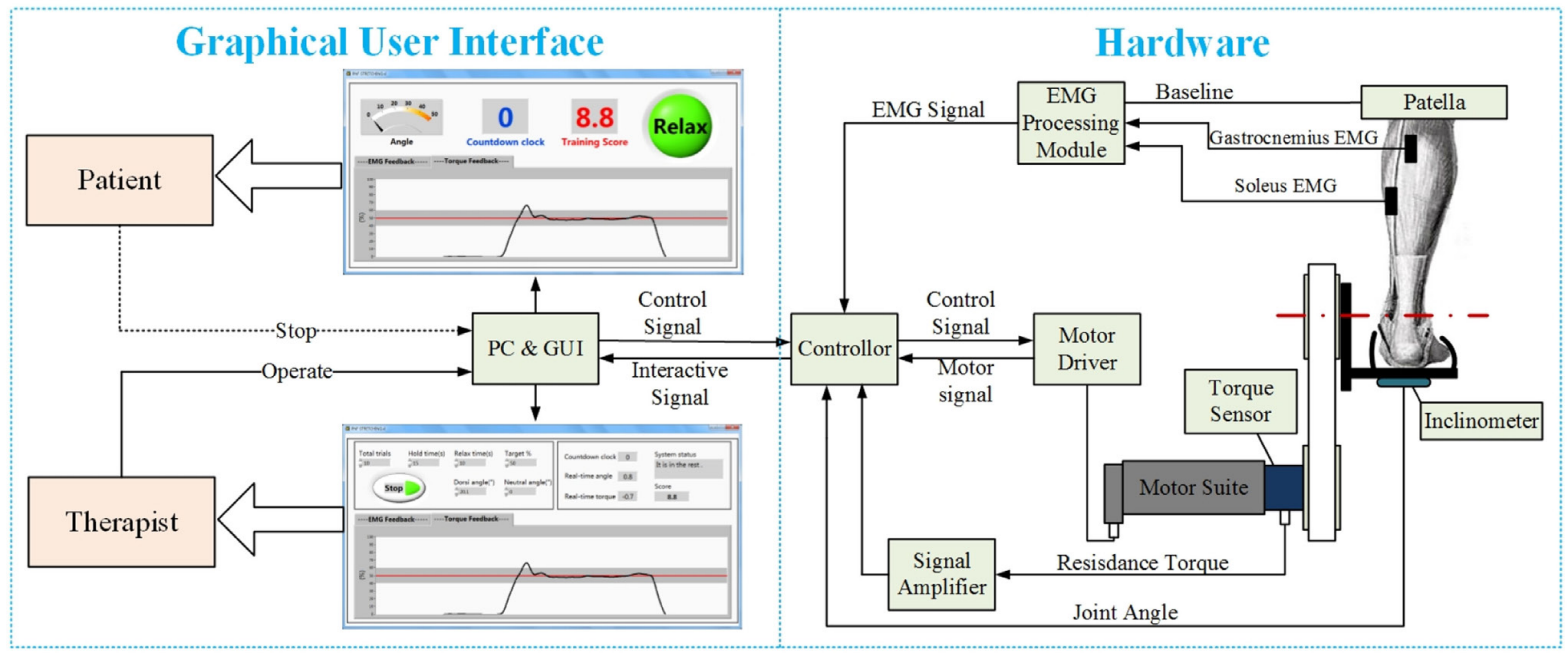
**The overall structure of the proposed PKU-RARS**. It is functionally divided into two parts: the graphical user interface and the hardware.

The PKU-RARS (shown in Figure 1) is a platform-based ankle–foot device for robotic rehabilitation performing PNF stretching on ankle plantar flexors spasticity (Zhou et al., 2015). The proposed device can be functionally divided into two parts: the graphical user interface and the hardware (shown in Figure 2). To ensure the safety while using the system, protection mechanisms on the control system and graphical user interface, mechanical stops, and emergency switches are redundantly designed in our system.

### 2.1 Apparatus Design

The proposed device is composed of an adjustable mechanical seat, a transmission mechanism, an adjustable sliding platform, an adjustable leg support, a robotic footplate, and a control cabinet. The height and position of each component is adjustable to ensure that the system can be used by people with different height and leg length, while ankle joint is aligned with the footplate rotation axis and knee flexed at the same degree during each training (shown in Figure 1). Since we adopt an active method, which requires the patients to perform their maximum force during the treatment, the hardware structure demands high mechanical rigidity to guarantee the patient and the device to be relatively still during training.

The footplate is driven by a motor suite (Dunkermotoren Gmbh) through a compliant belt transmission. An inline rotation encoder is installed for speed measurement. The rotation torque is measured by a uniaxial torque sensor (Transducer Techniques LLC.), which is mounted on the shaft. An inclinometer (CTL Components PLC.) is attached underneath the footplate for measuring joint angle. During the training, electromyography (EMG) signals from gastrocnemius and soleus muscles are recorded by a two-channel EMG system (Delsys Inc.). All signals are fed into the program through a USB Data Acquisition Card (National Instruments Inc.) with the sampling rate of 1 kHz. Raw torque and angle signals are low-pass filtered through a second order Butterworth filter with a cutoff frequency of 20 Hz. Raw EMG signals are bandpass-filtered through a fourth order Butterworth filter with cutoff frequency of 20 and 450 Hz. After full-wave rectifying, another fourth order Butterworth filter with cutoff frequency of 5 Hz is used. In order to provide a clear visual feedback for participants during training, moving average with a window length of two hundred sample points is applied to the filtered EMG signal. Processed data are presented on a customized graphical user interface developed in LabVIEW environment.

### 2.2 Control System

The control strategy of PKU-RARS is based on a hierarchical architecture comprising a low-level control layer and a high-level control layer. The low-level control consists of a closed-loop control for torque, velocity, and position. The specific low-level control mode is set up by the high-level control.

#### 2.2.1 Low-level Control System

**Figure 3:**
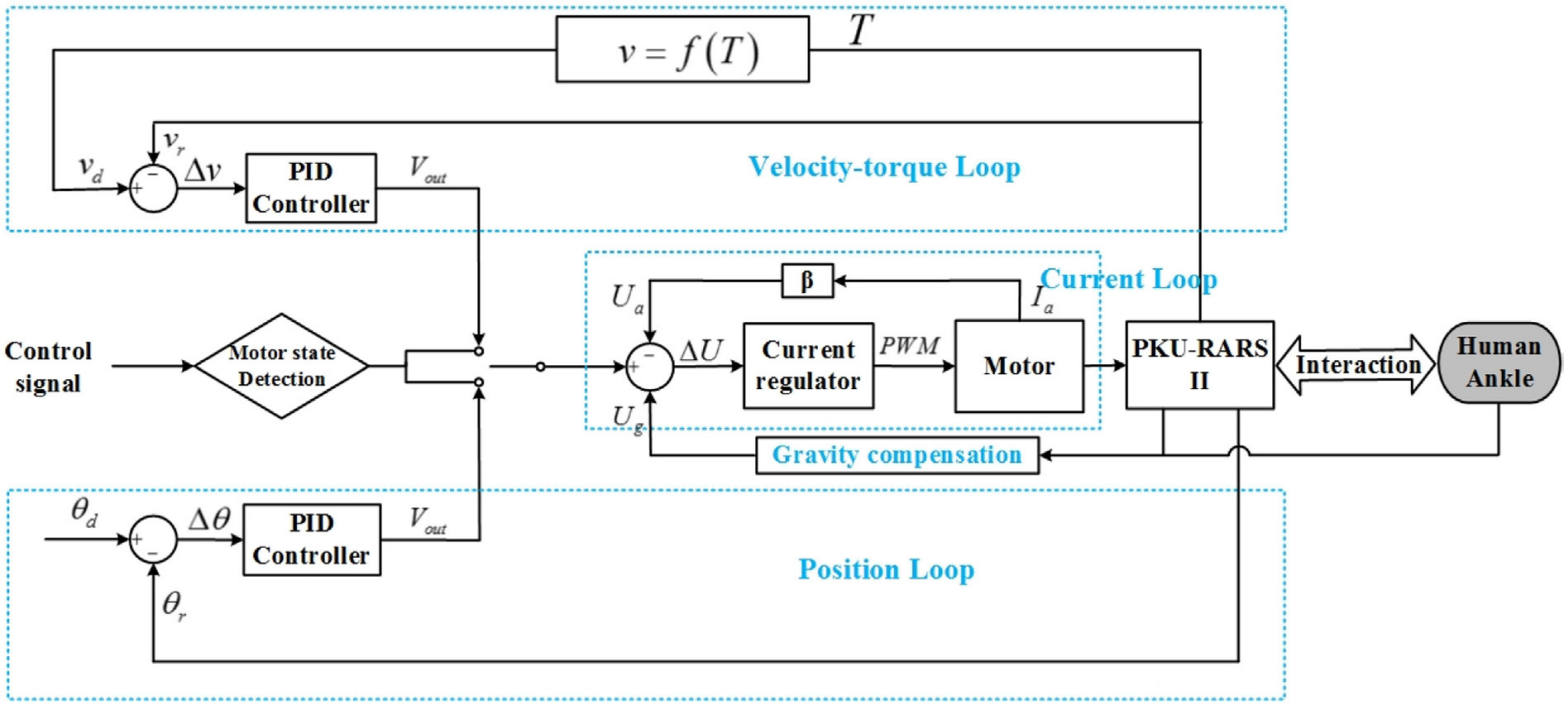
**The control framework of the proposed PKU-RARS**. It is a hierarchical architecture comprising a low-level control layer, which implements a basic torque, velocity, and position closed-loop control, and a high-level control layer, which outputs control signal to low-level controller and performs the execution of the whole specific rehabilitation protocol.

The low-level control system (see Figure 3) is implemented on a customized circuit where ARM-based STM32 is used as the processor. It includes a double closed-loop system with the current control as the inner-loop and the velocity–torque control or position control as the outer-loop. The outer-loop can be switched based on control signal from the high-level. In addition, gravity compensation is also completed in the low-level control to compensate the weight of the foot and footplate.

For the velocity–torque loop, the velocity of robotic footplate is set as inversely proportional to the resistance torque (Zhang et al., 2002; Gao et al., 2011) and is calculated by

(1) $$v=C_{1}+\frac{C_{2}}{\left|\tau_{res}\right|}$$

Although similar to Zhang et al. (2002), in the relationship of torque to velocity is inversely proportional, we do not set a specified peak resistance torque (a relatively small value) to decide a limited ankle ROM. In Zhang et al. (2002), the ankle joint is moved between two predefined positions, which do not cover the whole ankle ROM. Therefore, calf muscle can not be fully stretched into the extreme position of dorsiflexion where the spasticity and/or contracture is significant. In our treatment, extreme position of dorsiflexion is measured and used in the treatment.

Since the resistance torque is low when the ankle joint is at its neutral position, to improve the efficiency of the training, stretching is performed at a higher speed when the resistance is low. If, as in some cases, the resistance is high in the middle, the movement will also be slowed down accordingly. In our system, *C*_1_ and *C*_2_ are 0.05 and 7.5 respectively. The stretching velocity is set from 0.2 to 0.8 rpm to avoid inducing stretch reflex.

When the ankle joint reaches the desired position, the motor controller implements a double closed-loop where position closed-loop serves as the outer-loop and current closed-loop as the inner-loop (see Figure 3). In the proposed rehabilitation protocol, the patients are asked to performed isometric contraction in plantar flexion direction at the maximum dorsiflexion position.

#### 2.2.2 High-level Control System

**Figure 4:**
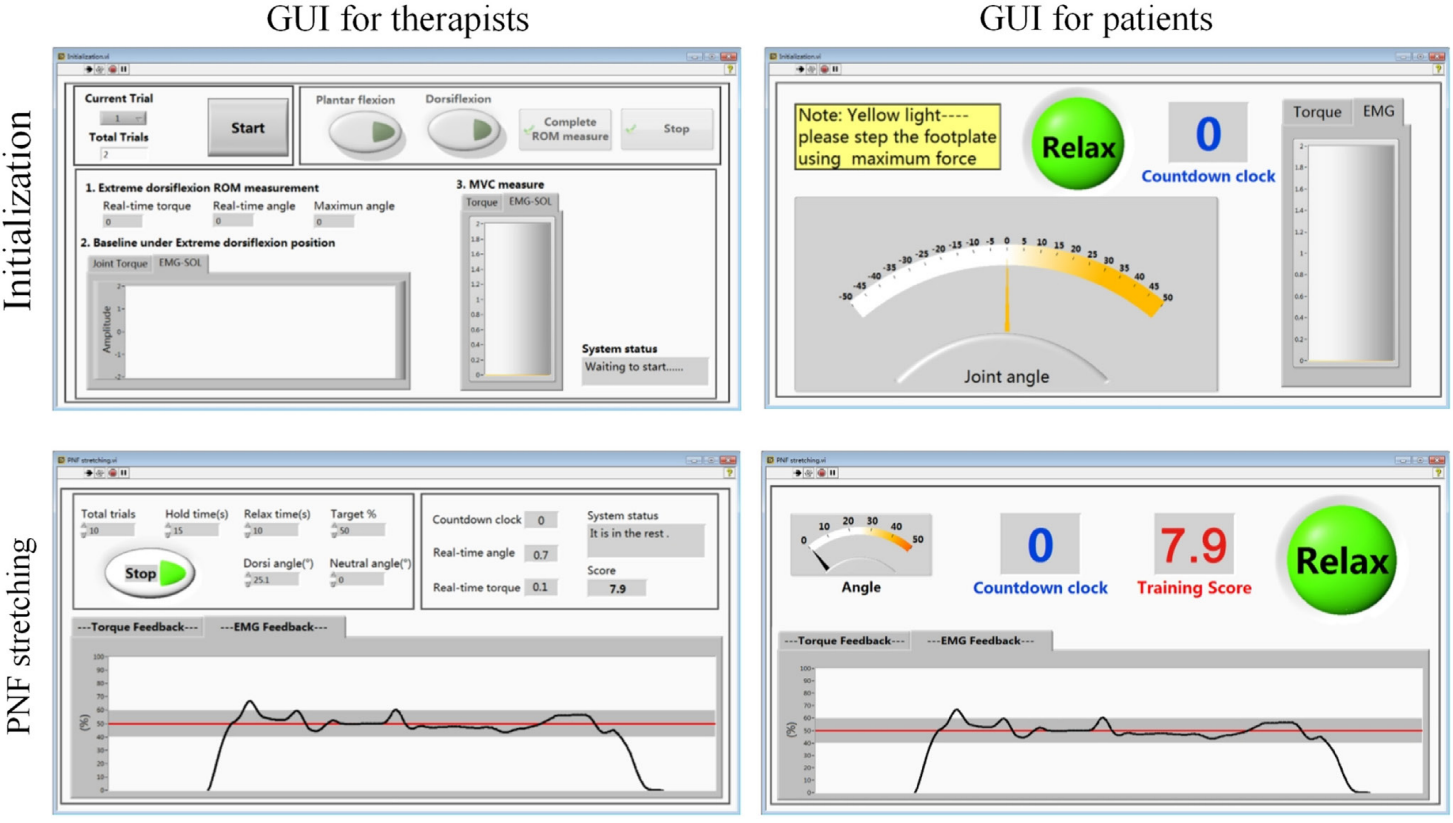
**The customized graphical user interface developed in the Labview environment**.

The high-level control system is implemented in a customized GUI developed in LabVIEW environment (see Figure 2). Based on the rehabilitation training protocol, the high-level control system will initiate and set the training parameters for the velocity–torque control loop or position control loop. Detailed protocol will be described in the next section. Figure 4 shows the interfaces for therapist and patients during training. More in-depth information of the GUIs designed for this work can be found in our publication (Zhou et al., 2015).

### 2.3 PKU-RARS Improvement and Rationale

Improving from the previous version (Zhou et al., 2015), the modified PKU-RARS has a more user-friendly mechanical design and human–machine interaction. Modifications were made by taking into account patients’ feedback of previous design. First, the suggestions included difficulty sitting in a static seat and getting into position when approaching from the side. These issues were addressed by fitting a mechanically rotatable seat that allowed the operator to rotate the seat after the patient sits down so that the patient can face the foot plate. Second, there were seven DOFs that needed to be adjusted to reach a suitable body position in the previous system, which was time-consuming and labor-intensive. Here we redesigned the robotic structure to streamline the redundant DOFs to four. Finally, a compliant belt transmission was embedded into the motor output shaft and the footplate instead of the direct drive. The compliant element can absorb the direct shock of motor to protect ankle joint and ensure safety. For the control system, gravity compensation was added in order to obtain a more stable and smoother performance and accurate joint torque measurement.

## 3 Methods

### 3.1 PNF-Based Therapy

The PNF technique was proposed by Kabat and Knott (1953) for the rehabilitation of polio patients with paralysis. It was reported that the PNF treatment can significantly improve flexibility, ROM, muscle strength, and activity daily living (ADL) function (Klein et al., 2002). Compared with passive stretching, PNF stretching is more effective (Funk et al., 2003). Thus, it has been extensively used for both rehabilitation training and athletic training (Marek et al., 2005).

PNF hold–relax method has been applied in the robotic system (Surburg and Schrader, 1997; Prentice and Voight, 2001). The mechanism of PNF has been explained by Sherrington’s laws of irradiation, successive induction, and reciprocal innervation. In human limbs, Golgi tendon organs (GTO) serve as proprioceptive sensory receptor organs and provide information about changes in muscle tension. In PNF stretching, a strong isometric contraction of the target muscle will trigger the GTO to lead to Golgi tendon reflex that can relax the target muscle to facilitate lengthening. Therefore, the patients actively contract ankle plantar flexors, allowing these muscles get further relaxed. The repetition of this process can facilitate patients to further contract and relax their spastic plantar flexors.

### 3.2 Participants

Seven poststroke patients (six males and one female) were recruited from the Department of Rehabilitation Medicine, First Hospital, Peking University. Their relevant characteristics are listed in Table 1. The inclusion criteria for these participants includes (1) at least 6 months post-stroke; (2) ankle spasticity in the paretic side; (3) able to walk with a cane or without any mechanical aid, and (4) can generate plantar flexion torque by activating the calf muscles. Exclusion criteria are having other severe disease, leg musculoskeletal injuries, and/or orthopedic surgeries on the leg. In addition, their unimpaired sides were regarded as the control group to reduce the intrinsic variability in different patient groups due to individual difference. All procedures were approved by the institutional review board of Peking University. Participants provided informed consent before the experiment.

**Table 1:** **Participant characteristics**.

No.	Age (year)	Gender (M/F)	Affected side (L/R)	Post injury duration (months)	Ankle MAS
1	79	M	L	67.7	2
2	41	M	L	76.9	2
3	73	M	R	43.3	2
4	42	M	R	50.0	2
5	51	M	R	47.7	3
6	40	M	L	18.6	2
7	76	F	R	30.0	3
Summary (mean ± SD)	57.4.6 ± 17.8	6M and 1F	4R and 3L	47.9 ± 18.4	2.29 ± 0.49

### 3.3 Treatment Protocol

**Figure 5:**
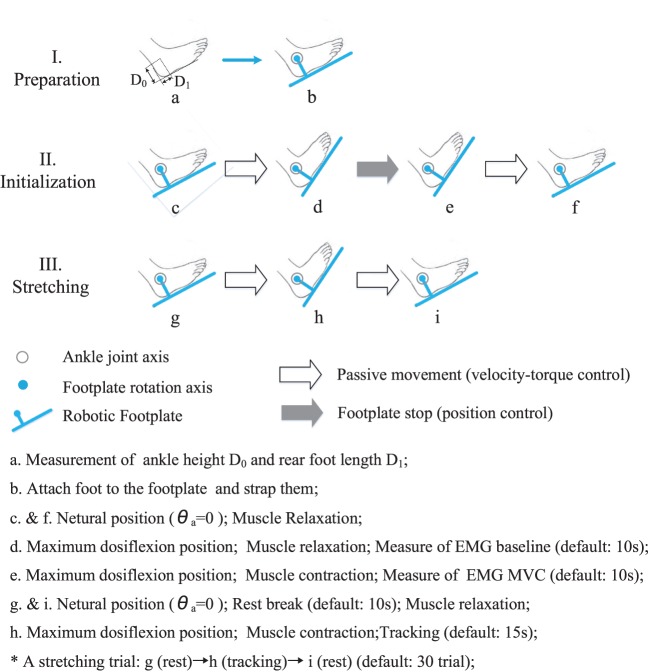
**The protocol of a PNF training session**. It mainly involves three steps, namely, preparation, initialization, and stretching. (a) Measurement of ankle height D0 and rear foot length D1; (b) attach foot to the footplate and strap them; (c and f) neutral position (θ_a_ = 0); muscle relaxation; (d) maximum dosiflexion position; muscle relaxation; measure of EMG baseline (default: 10 s); (e) maximum dosiflexion position; muscle contraction; measure of EMG MVC (default: 10 s); (g and i) neutral position (θ_a_ = 0); rest break (default: 10 s); muscle relaxation; (h) maximum dosiflexion position; muscle contraction; tracking (default: 15 s); *A stretching trial: g (rest) → h (tracking) → i (rest) (default: 30 trials).

The training was carried out three times a week (generally Monday, Wednesday, and Friday) and lasted for 3 months. Before and after each session of PNF training, a 2-min passive stretching between joint neutral position and maximum dorsiflexion position was performed to warm-up or relax the soleus and gastrocnemius muscles. Followed by warm-up, 30 trials of PNF stretching were performed. One PNF trial involved three major steps, namely preparation, initialization, and stretching. A schematic figure of the PNF protocol was presented (shown in Figure 5).

#### 3.3.1 Preparation

Participants were seated comfortably with their ankle aligned with the axis of the motor shaft and knee flexed at 30° (Zhou et al., 2015). The lower leg was strapped to the leg support, and foot was secured on the footplate by Velcro belts at the dorsal foot and the heel. The length of the lower leg, height of foot (distance from the bottom of the foot to the lateral malleolus), and foot size were measured in the first training session and were used for the same participants following sessions. After cleaning the skin, surface EMG electrodes were attached to the soleus muscle. The electrodes location was determined according to the recommendation of the SENIAM Protocol (Surface ElectroMyoGraphy for the Non-Invasive Assessment of Muscles).

#### 3.3.2 Initialization

After warm-up, the extreme position in ankle dorsiflexion was measured. To prevent injury, the dorsiflexion ROM angle was determined as the maximum angle that will not cause any pain. When the maximum dorsiflexion angle was reached, motor was shut down, and angle was recorded in the system. Participants’ soleus EMG and ankle torque during maximum voluntary contraction (MVC) in plantar flexion direction was measured three times (shown in the upper two subfigures of Figure 4). The peak value of MVC–EMG was recorded for normalization in the PNF stretching.

#### 3.3.3 Stretching

During the PNF stretching, the ankle joint was rotated from its neutral position to the extreme dorsiflexion position. Then, the subject performed isometric contraction with the soleus muscle activated and maintained the soleus EMG in the range 50 ± 10% MVC for 15 s. The target range and the duration of contraction were adjusted based on actual condition of different subjects and the rehabilitation phase. Processed EMG and target range were presented through a customized GUI interface for patient (shown in the bottom two subfigures of Figure 4). After the 15 s muscle activation, ankle joint was moved back to its neutral position to relax the muscle. The break between each PNF trial was 10 s and could be adjusted according to the participants’ needs. One typical training session was completed between 30 min and 1 h.

### 3.4 Outcome Analysis

For stroke patients, brain dysfunctions related to disease of the blood vessels supplying the brain can lead to the loss of muscle inhibition and cause spasticity. The PNF-based robotic rehabilitation might be able to improve properties of ankle joint and the triceps surae muscles, and further recover daily ambulation and functional activities. To investigate the effects of robotic PNF training, biomechanical and clinical tests were performed before and after the 3-month training. Biomechanical measurements include passive and active ankle ROM, ankle resistance torque during passive stretching, size of Achilles tendon. Clinical measurements include the Timed Up-and-Go (TUG), 10-m walking at comfortable walking speed, and fast speed.

#### 3.4.1 Biomechanical Measurements

##### 3.4.1.1 Passive Properties

**Figure 6:**
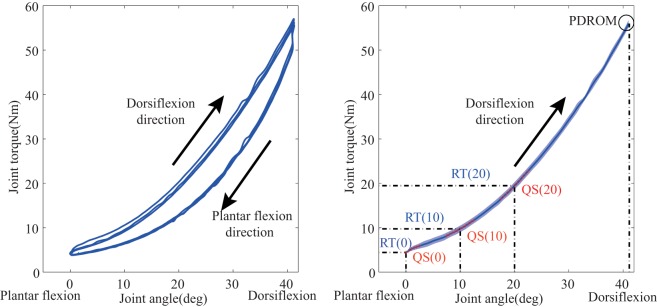
**Passive properties of ankle joint**. The left figure: hysteresis loop. Joint angle and resistance torque are measured in the passive movement (three times). Each hysteresis loop is divided into 2 limbs, the ascending limb for dorsiflexion direction movement and the descending limb for plantar flexion direction movement. The right figure: the averaged torque-angle curve (the shade denotes the SD). It is used to calculate resistance torque (RT) and the quasi-static stiffness (QS) under three specified joint angle (0°, 10°, and 20°). QS is calculated as the slope of the regression curve to fit 11 data points [5 points before and after (red point)] around a specific joint angle.

To test the ankle quasi-static stiffness, joint ROM and resistance torque were measured while the ankle was passively moved by the motor. Angle and its corresponding resistance torque were plotted as a hysteresis loop as showed in Figure 6. Each hysteresis loop was divided into 2 limbs, the ascending limb indicating the ankle was moved in dorsiflexion direction, and the descending limb indicating the movement in plantar flexion direction. Joint stiffness is usually defined as ∆*T*/*∆θ*, where ∆*T* is the passive torque increment when the ankle angle changed (∆*θ*) (Burstein and Wright, 1994). In this study, a regression curve was first plotted based on 11 torque value (5 data points before and after training) at a specific ankle angle. For further statistical analysis, we compared the resistance torque and the quasi-static stiffness under three specific joint angles (0°, 10°, and 20°). In addition, passive dorsiflexion ROM (PDROM) was defined as the dorsiflexion angle corresponding individual maximum resistance torque. Those measurements were only conducted in dorsiflexion since the proposed system was aimed at treating the stretching flexor muscles. Average parameters of the first week and the last week were used in contrastive analysis.

##### 3.4.1.2 Active Properties

Active properties included joint torque during maximum voluntary contraction (TMVC) and training score (TS) during the PNF stretching (Zhou et al., 2015). TMVC was defined as active plantar flexion torque under the extreme dorsiflexion angle. TS (scaled to 0–10) reflected the muscle control ability in ankle plantar flexors (Zhou et al., 2015). Average parameters of the first week and the last week were used in contrastive analysis. PNF as an active intervention might improve those active properties with respect to CPM.

##### 3.4.1.3 Achilles’s Tendon Properties

**Figure 7:**
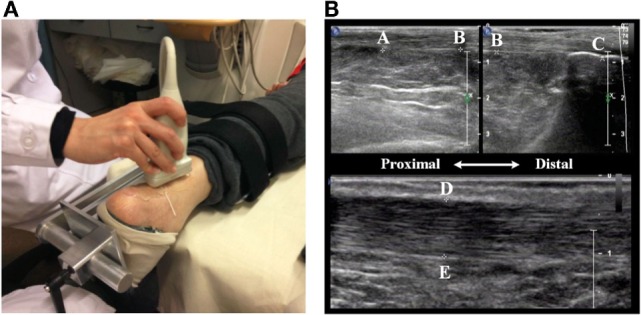
**Ultrasonic measurement of AT**. **(A)** The ankle is held at the natural position (90° between the leg and the foot) using a fixed apparatus. **(B)** The length of the AT is defined from the muscle–tendon junction (MTJ) (Point A) to its insertion into the calcaneus notch (Point C). The thickness of the AT is defined the distance between two points (D and E).

Spastic hypertonia may cause size changes for AT (Gao et al., 2011). To test whether the PNF training could affect the size of AT, AT length and thickness were measured at both ankles during pretest and posttest by using Philips IU22. Figure 7A shows the setup and method used for measuring the size of AT. During the measurement, participants were relaxed in prone position with their ankle held in the neutral position. The probe applied with ultrasound transmission gel was placed perpendicular to the skin with moderate pressure and moved smoothly along the crest of the AT without inducing tendon reflex.

AT length (shown in the top figure of Figure 7B) was measured from the muscle–tendon junction (MTJ) (Point A), where AT (or free tendon) divided into soleus aponeurosis and gastrocnemius tendon, to its insertion into the calcaneus notch (Point C). A tissue string was placed on the skin surface as a marker (Point B), around the level of the tip of the medial malleolus. The marker divided the length of the AT into two short segments due to the limit of probe size. These two segments were captured in two parallel windows using dual mode function. Their sum is considered as the total length of the AT. To measure the thickness of the AT, the probe was applied to the AT from posterior-to-anterior direction. On the sagittal plane of the AT, a line vertical to the fiber of the AT was drawn at from tip of the medial malleolus (shown in the bottom figure of Figure 7B). This line intersects with AT aponeurosis at two points (Point D and Point E), the distance between Point D and E was measured as the thickness of the AT. To minimize the measurement error, the averaged value from three repetitions was used for further analysis.

#### 3.4.2 Clinical and Functional Measurement

Functional evaluations included 10-m walking at different speed and the Timed Up-and-Go test (TUG). Walking speeding is an important indicator of locomotion function and is also important in daily activity. Also, 10-m walking was measured at two different speeds by using GAITRite^®^ walkway system (CIR Systems, Inc.). At first, subjects were asked to walk five times at a normal speed, which they think is their optimum speed in daily ambulation. Then, another five trials of fast walking was performed at the fastest speed without losing stability. TUG is a simple test used to assess a person’s mobility and requires both static and dynamic balance. It uses the time that a person takes to rise from a chair, walk 3 m, turn around, walk back to the chair, and sit down. During the test, the person is expected to wear their regular footwear and use any mobility aids that they would normally require.

#### 3.4.3 Statistical Analysis

Repeated measurements were used to analyze the response variables including all biomechanical, clinical, and functional outcome indicators. All variables were processed and calculated using MATLAB^®^ (The Mathworks, Inc.). Paired *t*-tests were conducted to study the differences between groups with a significance level at 0.05. Adjustments were made if a violation of sphericity was found. All statistics were done by using SPSS (Statistical Product and Service Solutions, IBM).

## 4 Results

### 4.1 Biomechanical Changes

#### 4.1.1 Decreased Passive Resistance Torque

**Figure 8:**
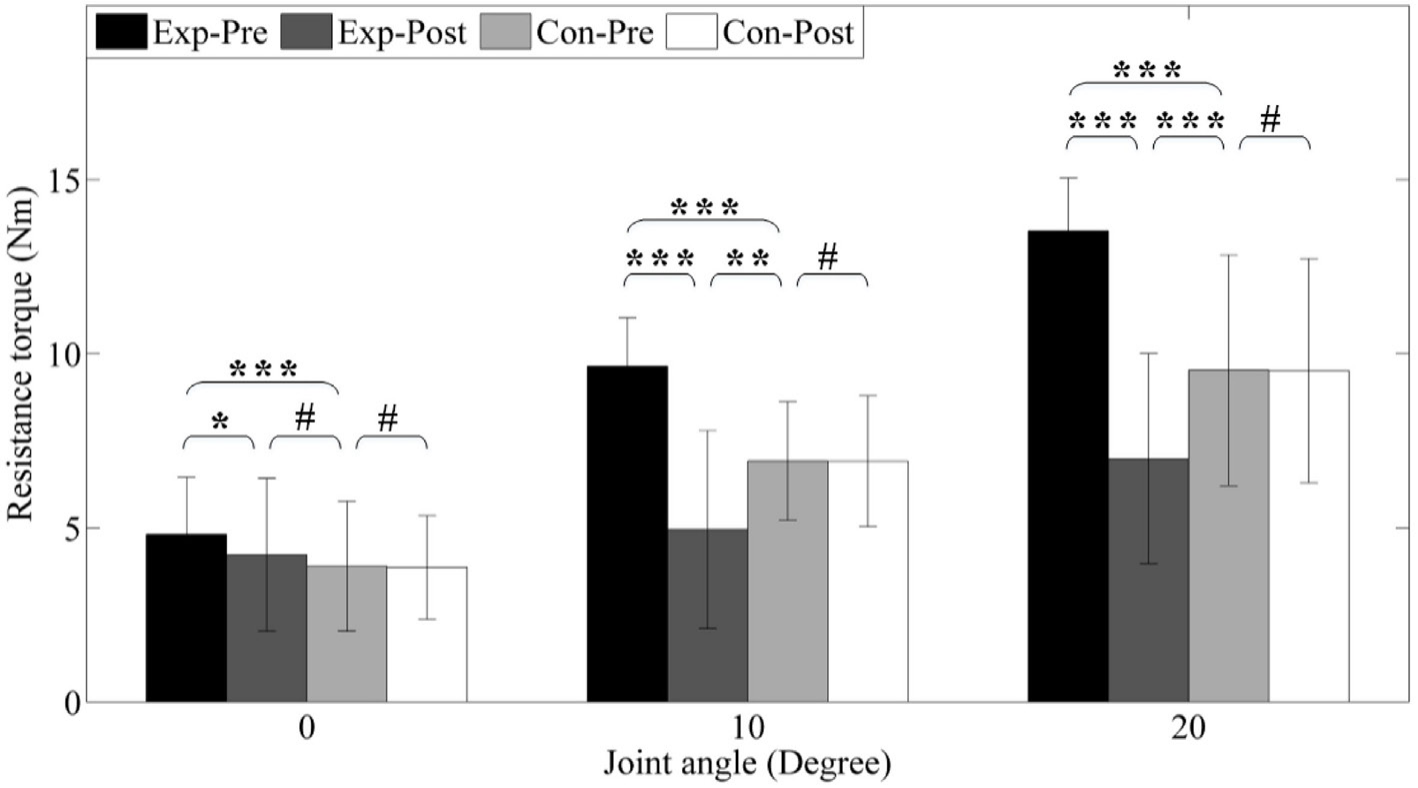
**Resistance torque under different joint angle level**. *Exp-* and *Con-* represent experimental group and control group, respectively. *-Pre* and *-Post* represent before and after the 3-month interventions (* stands for a significance level, and ^#^ represents no significant difference).

Figure 8 shows the resistance torque of each group before and after interventions. Before interventions, the experimental group shows significant higher (*p* < 0.001) resistance torque (4.81 ± 0.82, 9.64 ± 0.35, and 13.53 ± 0.50 Nm) under different dorsiflexion angle level (0°, 10°, and 20°) compared to the control group (3.90 ± 0.93, 6.92 ± 0.43, and 9.53 ± 1.10 Nm) due to ankle spasticity.

After interventions, resistance torques in the experimental group significantly reduced to 4.22 ± 0.11, 4.95 ± 0.71, and 6.99 ± 1.00 Nm (*p* < 0.05, *p* < 0.001, and *p* < 0.001), but no alteration is found in the control group (all *p* > 0.05) due to no intervention. Under neutral position (0°), the resistance torque of control group is significantly reduced, but it is still higher than the control group (*p* = 0.056). However, under dorsiflexion angle 10° and 20°, we can find that these parameters post-interventions are lower than the control group (*p* < 0.01 and *p* < 0.001). In addition, PDROM increases from 32.28 ± 4.22° to 42.13 ± 5.95° (*p* < 0.01).

#### 4.1.2 Decreased Quasi-Static Stiffness

**Figure 9:**
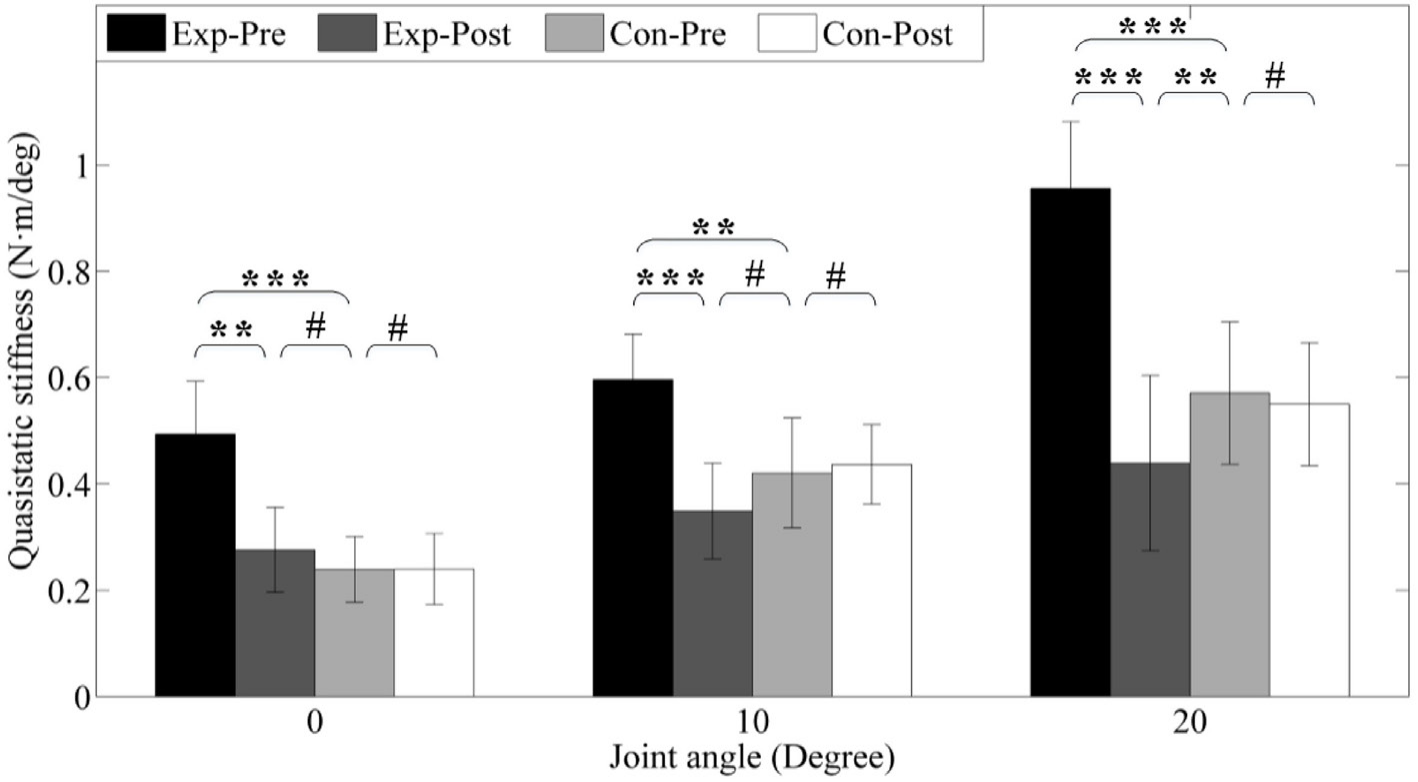
**Quasi-static stiffness under different joint angle level**. *Exp-* and *Con-* represent experimental group and control group, respectively. *-Pre* and *-Post* represent before and after the 3-month interventions (* stands for a significance level, and ^#^ represents no significant difference).

To further investigate the passive properties of the ankle joint, quasi-static stiffness was compared between pretest and posttest and between groups. The results are presented in Figure 9. Before interventions, the experimental group presents higher stiffness (0.49 ± 0.09, 0.60 ± 0.08, 0.96 ± 0.08 Nm/°) under different dorsiflexion angle level (0°, 10°, and 20°) than the control group pre-interventions (0.24 ± 0.06, 0.42 ± 0.09, 0.57 ± 0.09 Nm/°) due to ankle spasticity, and the differences are highly significant (*p* < 0.001, *p* < 0.01, and *p* < 0.001). After interventions, stiffness of the experimental group are 0.28 ± 0.08, 0.35 ± 0.08, and 0.49 ± 0.11 Nm/°, which are significantly reduced (*p* < 0.01, *p* < 0.001, and *p* < 0.001), but there is also no alteration for control group (all *p* > 0.05) due to no intervention. The improvement of experimental group compared with the control group is similar to the result of the passive resistance torque of ankle joint, and the detailed values are presented in Figure 9.

#### 4.1.3 Improvement of Active Properties

**Figure 10:**
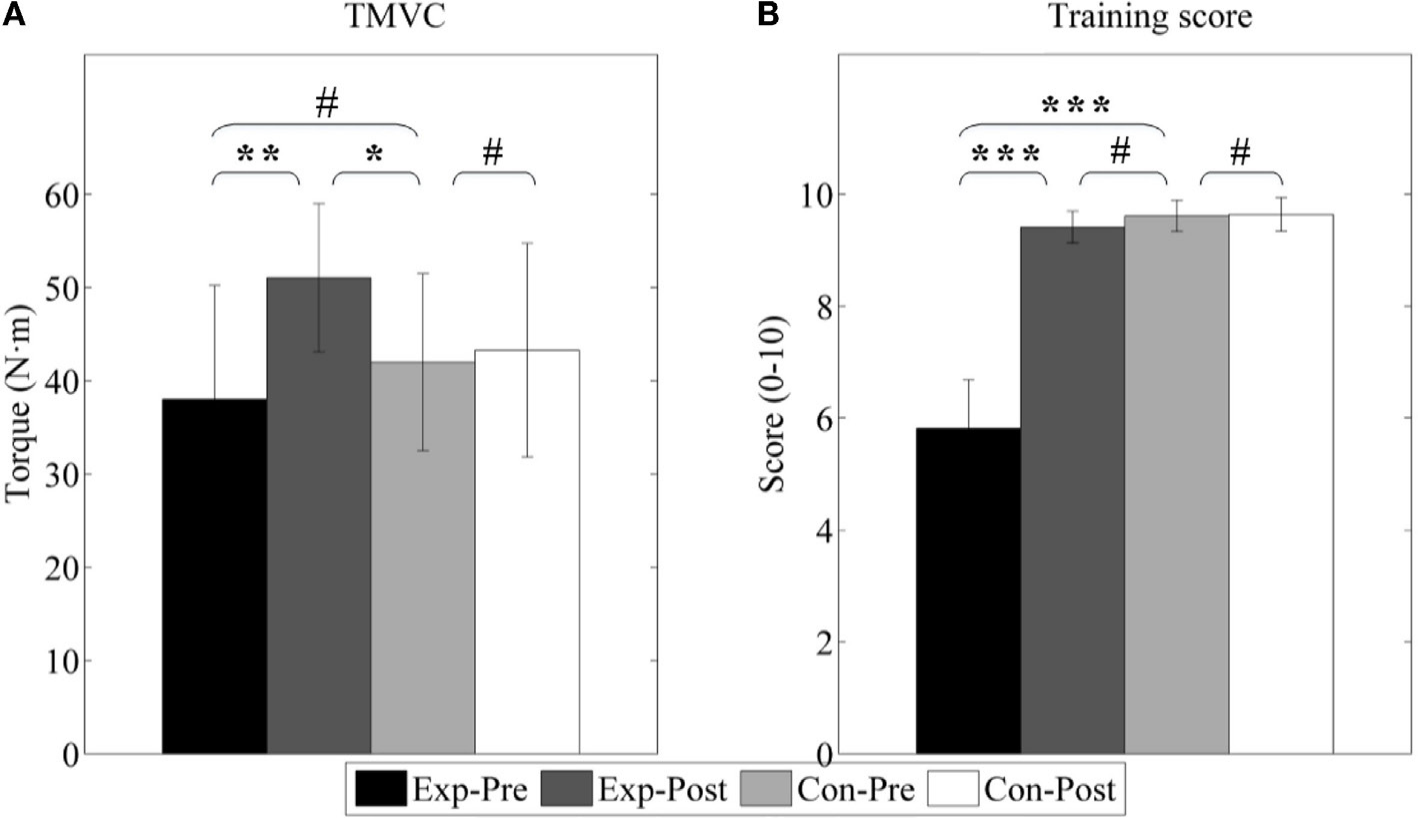
**Active properties of ankle joint**. **(A)** Joint of maximum voluntary contraction (TMVC); **(B)** train score during PNF stretching (TS). *Exp-* and *Con-* represent experimental group and control group, respectively. *-Pre* and *-Post* represent before and after the 3-month interventions (* stands for a significance level, and ^#^ represents no significant difference).

Figure 10 shows the results of the maximum voluntary contraction torque (TMVC) and training score (TS). TMVC of experimental group is slightly lower than control group (*p* > 0.05) although it is not significant, and TS is largely poor (*p* < 0.001). We can also find after interventions TMVC increases from 38.03 ± 12.21 to 51.04 ± 7.95 Nm (*p* < 0.01) as well as TS from 5.81 ± 0.87 to 9.41 ± 0.29 (*p* < 0.001). Furthermore, TMVC strength is found to be greater than that of the control group (41.99 ± 9.49 Nm, *p* < 0.05). And, TS is nearly to that of the control group (9.61 ± 0.28, *p* > 0.05).

#### 4.1.4 Shortening of AT

**Figure 11:**
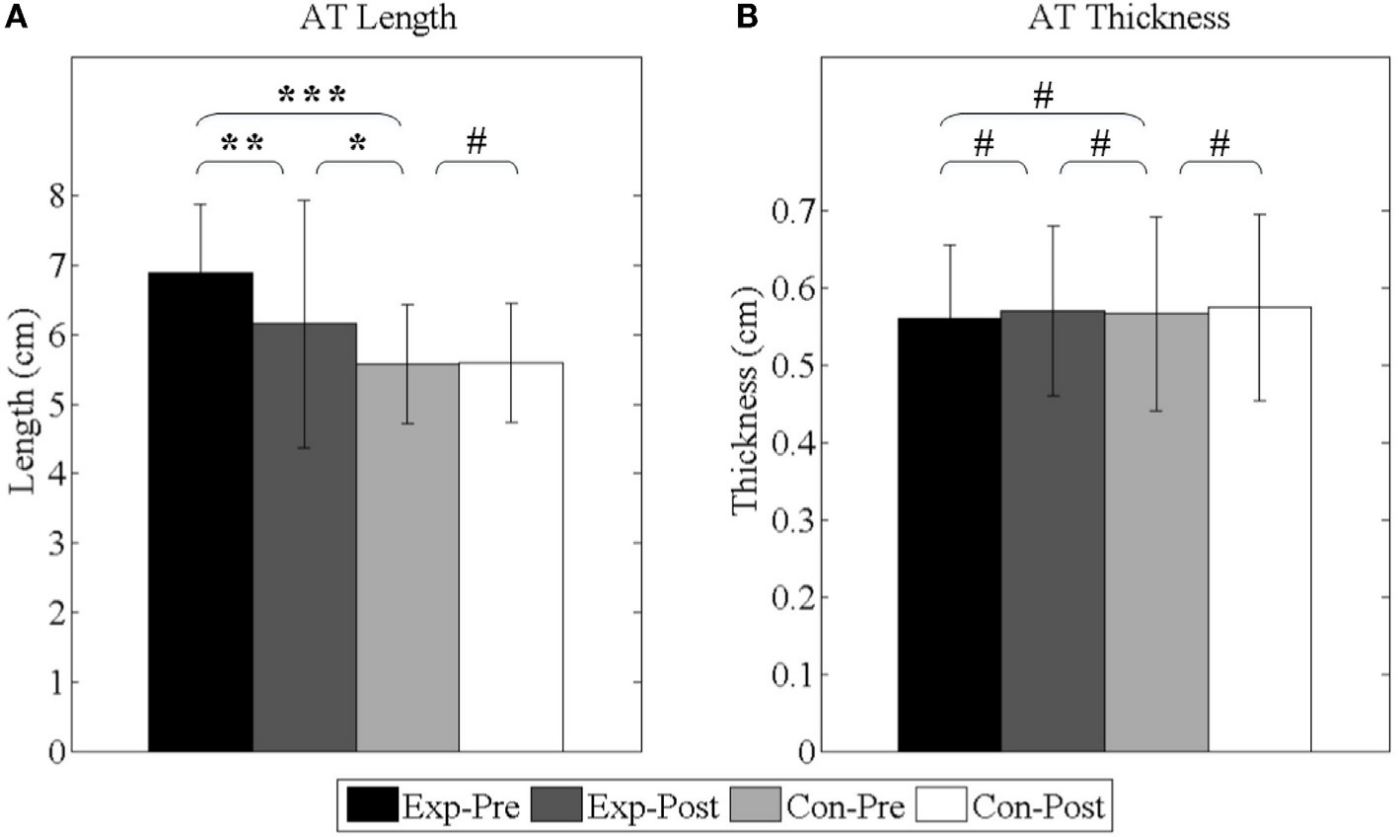
**Achilles’s tendon properties**. **(A)** The length of Achilles’s tendon; **(B)** the thickness of Achilles’s tendon. *Exp-* and *Con-* represent experimental group and control group, respectively. *-Pre* and *-Post* represent before and after the 3-month interventions (* stands for a significance level, and ^#^ represents no significant difference).

As shown in Figure 11, before interventions, the lengths of experimental group and control group are 6.89 ± 1.00 and 5.57 ± 0.86 cm, respectively, and the elongation is averagely 1.32 cm (*p* < 0.001). After interventions, the length of experimental group is shortened to 6.15 ± 1.18 cm (*p* < 0.01) and still longer than control group (*p* < 0.05). Additionally, the thicknesses of experimental group and control group are 0.56 ± 0.10 and 0.57 ± 0.13 cm, respectively, before interventions (*p* = 0.73). After interventions, the thickness of the experimental group has no significant difference (*p* = 0.28).

### 4.2 Clinical and Functional Outcomes

**Figure 12:**
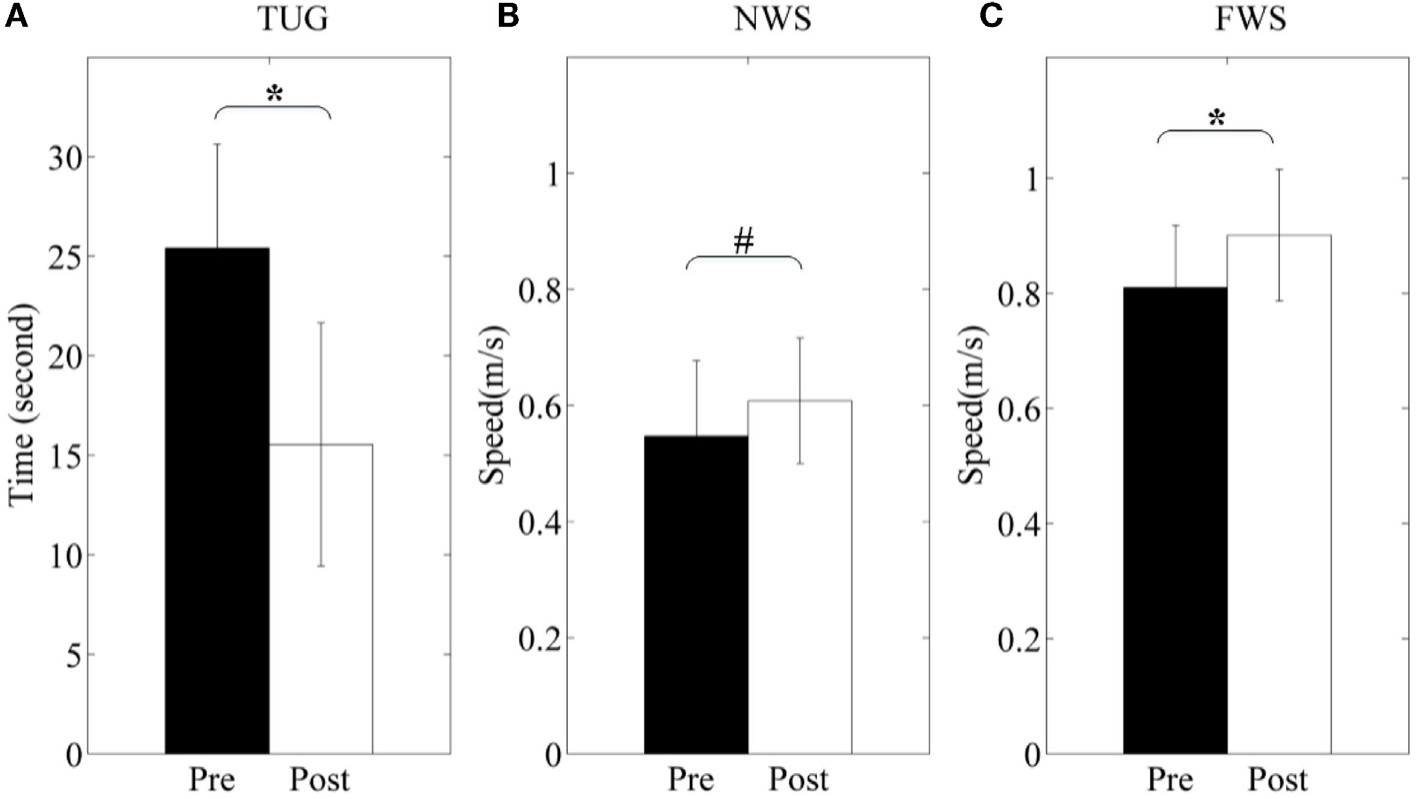
**Clinical and functional evaluation**. **(A)** Time Up-and-go (TUG); **(B)** normal walking speed (NWS); **(C)** fast walking speed (FWS). Pre and Post represent before and after the 3-month interventions (* stands for a significance level, and ^#^ represents no significant difference).

The participants show functional improvements in terms of TUG and walking speed before and after 3-month rehabilitation (shown in Figure 12). TUG scores are from 25.4 ± 15.3 to 15.7 ± 6.1 (*p* < 0.05). Normal walking speed and fast walking speed are from 0.547 ± 0.130 to 0.607 ± 0.108 m/s (*p* = 0.053) and from 0.810 ± 0.108 to 0.901 ± 0.114 m/s (*p* < 0.05), respectively. Particularly, the only female participant hardly finished the fast walking measure before training, but she had no difficulty after training.

## 5 Discussion

In this study, we developed a modified platform-based robotic ankle–foot rehabilitation system (PKU-RARS) and investigated the effects of PNF-based robotic rehabilitation on ankle plantar flexors spasticity. In comparison with some previous platform-based ankle devices (Zhang et al., 2002; Deutsch et al., 2007; Homma and Usuba, 2007; Cordo et al., 2008; Burdea et al., 2013), PKU-RARS was designed with the considerations of convenient wearability, interactive safety, and interface friendliness. Moreover, we combined a neurorehabilitation method with a robotic rehabilitation system. Different from existing CPM-based robotic treatments discussed in those studies (Singer et al., 2002; Zhang et al., 2002; Homma and Usuba, 2007; Gao et al., 2011), PNF is a neurodevelopmental technique widely used by physical therapists. Motor elements and developmental components of complex motor patterns are chosen and reeducated through training the fundamental skills of limbs control, stability, and coordination. The robotic system integrated with PNF is based on the basic neurophysiologic and kinesiologic principles of the sensory–motor system. In the proposed robotic system, proprioceptive sensory receptor (the Golgi tendon organ) and Golgi tendon reflex are used in our robotic treatment. Compared with the PNF therapeutic exercise, the robotic PNF treatment can provide feedback between the human–machine interactions. Patients can have a visual feedback, while their limbs have a kinematic and dynamic interaction with the robot. They not only know their joint properties quantitatively but also control their limbs according to the visual feedback through the brain. Therefore, the closed-loop between brain and limb and the closed-loop between human and robot are integrated. The active participation during the training can improve compliance and initiative of patients and make them more interested in attending a long-term course. Although this study did not include the result without visual feedback, we compared some indexes with the robotic CPM therapy that do not require patients’ active participation and visual feedback. The subject performed isometric contraction under the extreme dorsiflexion position in PNF through proprioceptive response. The proposed system stretched the ankle forcefully and safely to its extreme positions where spasticity and contracture are significant, but the previous passive method used in Gao et al. (2011) was stretching in joint ROM according to a fixed torque threshold.

The pathological symptoms of ankle spasticity have been extensively studied including the reduced ROM, the increased resistance torque, and the higher quasi-static stiffness compared with the healthy side. The results in this paper (biomechanical properties of experimental and control group pre-intervention) are in agreement with previous studies (Harlaar et al., 2000; Singer et al., 2002). Several studies reported positive effects of CPM on biomechanical measurement (Zhang et al., 2002; Homma and Usuba, 2007; Gao et al., 2011). Zhang et al. (2002) reported that dorsiflexion range increased from 11.9° to 16.5° at the same torque (10 Nm) after stretching. Gao et al. (2011) showed that the resistance torque was 11.9 ± 3.2 and 10.5 ± 3.3 Nm (*p* = 0.009), and the stiffness was 0.60 ± 0.13 and 0.49 ± 0.14 Nm/° (*p* = 0.013) at 20° dorsiflexion, pre- and post-intervention, respectively. Homma and Usuba (2007) described that the dorsiflexion angle increases by 5°. In our study, the rehabilitation with PNF decreased stiffness at 20° from 0.96 ± 0.08 to 0.49 ± 0.11 Nm/°, and PDROM was from 32.28 ± 4.22° to 42.13 ± 5.95° (*p* < 0.01). The results indicate that the proposed PNF-based method may be more effective than the passive stretching on ankle rehabilitation in the view of passive biomechanical characteristics.

Significant AT length shortening was observed after the proposed robotic rehabilitation with PNF. Previous study demonstrated that spastic muscles had shorter muscle fascicles and longer AT compared to that of unimpaired control group (Harlaar et al., 2000). The response to the joint movement stimulus might lead to elongation of muscle fascicles, which is accompanied by increment of the sarcomere length and/or increment of sarcomere numbers in series (Williams and Goldspink, 1978). Although muscle length is not measured in this study, we proved that muscle length is increased through the shortening of AT length after 3-month training. Such result could not be found in only repeated passive stretching for poststroke patients (Gao et al., 2011). Therefore, it can further demonstrate the effectiveness of the proposed PNF-based rehabilitation.

The relaxation of the triceps surae muscle may be benefited from the combined effect of biomechanical changes in muscle and AT. In addition, potential changes in aponeurosis such as decrease of quasi-static stiffness can also lead to relaxation of the calf muscle (Kubo et al., 2001), but we are unable to prove this element in this study.

Ankle plantar flexors are quite requisite in daily ambulation. The lack of push-off can lead to an insufficient forward propulsion, a decrease of swing initiation on the ipsilateral side, and a decrease of swing phase on the contralateral side (Neptune et al., 2001). Maynard et al. (2005) found that passive stretching might not improve gait significantly, while our results of active properties could demonstrate that the proposed method could enhance the muscle strength and obtain better control performance. Therefore, it may improve walking speed and gait cycle’s symmetry of stroke patients through gaining better control of the push-off and swing. In addition, clinical and function evaluation can also provide an evidence of improved gait patterns, which may be induced by the biomechanical changes of joint and muscle – not only the relaxation of the calf muscle but also the active biomechanical properties.

In our previous study (Zhou et al., 2015), we showed the feasibility of the proposed robotic ankle–foot system in the rehabilitation of plantar flexors spasticity for the poststroke patients. Preliminary evaluations were performed in the passive and active biomechanical properties of ankle joint after a 6-week PNF-based intervention. In order to further demonstrate the effectiveness of PNF-based rehabilitation for plantar flexors spasticity, current study carried out a long-term (3-month) intervention for seven post-stoke patients with hemiplegic spastic ankles. In the outcome evaluation program, except for those previous indicators, we further investigated the changes of the AT properties through ultrasonic technique to indirectly study the change of muscle fascicles. And more important than that, clinical and functional evaluations included TUG, and 10-m walking speed measure were analyzed, which might be indicators of the improvements of the musculoskeletal properties. Statistical analysis was performed on those experimental results. In addition, we also improved the robotic ankle–foot system according to experiences in clinical experiments and feedback from patients and therapists. Those modifications include mechanical seat, robot structure, and control system, which are designed for better operating, long-term reliability, and human–robot interaction safety. Those improvements of PKU-RARS could benefit the interaction between patients and therapists.

This study provides some evidences that the PNF-based robotic system could potentially be used as an effective tool in clinical rehabilitation for ankle plantar flexors spasticity. However, there are several limitations in this study. First, this study involves a relatively small sample of patients in which there is only one female. Gender may play an important role in determining joint spasticity. Second, CPM training is widely used in robotic studies and rehabilitation device. In this paper, compared CPM training data were obtained from previous studies. In the future, we will further carry out experiments on more subjects and recruit corresponding poststroke survivors as the CPM training group. In addition, we will try more clinical treatment methods integrated with robotics for stroke rehabilitation, such as other neurophysiologic techniques, motor learning techniques, or cognitive therapeutic exercise (Morreale et al., 2016).

## 6 Conclusion

This paper demonstrated that the poststroke patients with ankle plantar flexors spasticity show improvements in biomechanical, clinical, and functional measurements after the proposed PNF-based robotic rehabilitation. It can reduce tightness of the triceps surae muscles and joint quasi-static stiffness under different joint level and increase the passive ROM. We also proved the passively lengthening of muscle fascicle or sarcomere through the change of AT length. In addition, the robotic training may improve walking speed and gait cycle’s symmetry through gaining better control of the push-off and swing. The newly designed robotic device is superior in the rehabilitation treatment due to the modifications on the previous system. To conclude, the proposed PNF-based robotic rehabilitation with visual feedback may serve as a promising method for stroke rehabilitation.

## Author Contributions

QW, KW, and FG designed research; ZZ, YS, NW, KW, and QW performed research; ZZ and YS analyzed data; ZZ and QW wrote the paper.

## Conflict of Interest Statement

The authors declare that the research was conducted in the absence of any commercial or financial relationships that could be construed as a potential conflict of interest.
